# Disappointment and regret enhance corrugator reactivity in a gambling task

**DOI:** 10.1111/psyp.12371

**Published:** 2014-10-24

**Authors:** Yin Wu, Luke Clark

**Affiliations:** a Behavioural and Clinical Neuroscience Institute, Department of Psychology, University of CambridgeCambridge, UK; b Centre for Gambling Research at UBC, Department of Psychology, University of British ColumbiaVancouver, British Columbia, Canada

**Keywords:** Decision making, Gambling, Counterfactual thinking, Regret, Disappointment

## Abstract

This study investigated how the corrugator and zygomaticus respond to decision outcomes (i.e., gains and losses). We used a gambling task in which participants were presented with obtained followed by non-obtained outcomes. Activity at the corrugator site was sensitive to decision outcomes, such that higher obtained losses (disappointment) and higher non-obtained gains (regret) both heightened corrugator reactivity. Activity at the zygomaticus site was not responsive to obtained or non-obtained outcomes, but did show sensitivity to emotional images in the same participants, in the form of a positive linear relationship with self-reported emotional valence. Corrugator activity was negatively related to emotional valence. The findings indicate the sensitivity of corrugator to objective decision outcomes and also counterfactual comparisons, highlighting the utility of facial electromyography in research on decision making and gambling behavior.

Facial electromyography (fEMG) is a useful tool for studying affective processes by measuring activity on skin above specific facial muscle sites in response to emotional probes. Using standardized affective pictures, Lang, Greenwald, Bradley, & Hamm (1993) showed that appetitive images increased muscle reactivity in the zygomaticus major region (i.e., the smiling muscle), while unpleasant pictures heightened response in the corrugator supercilii region (i.e., the frowning muscle). Subsequent work described a negative linear relationship between corrugator response and emotional valence, and a positive linear relationship between zygomaticus reactivity and emotional valence, using various types of affective stimuli including pictures, sounds, and words (Bradley & Lang, 2000; Larsen, Norris, & Cacioppo, 2003). In a combined fEMG and functional magnetic resonance imaging study, corrugator responses to negative images were associated with greater activity in the amygdala and a concurrent decrease in ventromedial prefrontal cortex activity (Heller, Lapate, Mayer, & Davidson, 2014).

There is increasing interest in investigating psychological mechanisms underlying social and moral decision making using fEMG. For example, unfair offers in the ultimatum game increased levator labii reactivity, a facial muscle region under the nose that is also responsive to bitter tastes and basic disgust, suggesting that unfair financial distributions may be “morally disgusting” (Chapman, Kim, Susskind, & Anderson, 2009). Unfair offers that elicited stronger levator responses were more likely to be rejected. Scenarios depicting different types of moral violations enhanced fEMG activity at levator, zygomaticus and corrugator sites, the strength of which was correlated with subsequent moral judgment (Cannon, Schnall, & White, 2010). Induced disgust also increased facial muscle reactivity to images depicting moral themes (Whitton, Henry, Rendell, & Grisham, 2014). Taken together, these findings highlight the sensitivity of facial muscle activity to social and moral decision making. Perhaps surprisingly, little is known about how the facial muscles react to decision outcomes (i.e., gains and losses) outside of a social context. In line with zygomaticus sensitivity to positive affect (Lang et al., 1993; Larsen et al., 2003), one study reported that monetary wins heightened zygomaticus response relative to losses, in the context of a competition task involving third-party arbitration decisions (Bediou, Mohri, Lack, & Sander, 2011).

The purpose of the present study was threefold. First, we aimed to validate the response patterns at the zygomaticus and corrugator sites to objective winning and losing outcomes in a gambling task. We hypothesized that winning outcomes would heighten zygomaticus activity compared to losses, whereas the loss outcomes would increase corrugator activity as a function of loss magnitude.

Second, we were interested in how activity at these facial muscles responds to counterfactual comparisons, that is, the mental processes by which people consider salient alternatives to the events that actually occurred (“what might have been”). Previous research has shown that the subjective ratings of gambling outcomes are affected by the outcome obtained but are also moderated by the presentation of non-obtained outcomes of nonselected options (see Epstude & Roese, 2008, for a review). An upward counterfactual refers to the comparison of an obtained outcome against a more desirable alternative, which typically intensifies negative affect and is termed *regret*. On the other hand, a downward counterfactual refers to the comparison against a less desirable alternative, heightening positive affect and is termed *relief*. Neuropsychological and brain imaging studies have identified key nodes in the emotional brain, including orbitofrontal cortex and striatum (Camille et al., 2004, 2010; Coricelli et al., 2005; Steiner & Redish, 2014), as being associated with the processing of counterfactual comparisons. Event-related potential (ERP) studies have investigated the temporal characteristics of these processes, showing that the P300, a late ERP component indexing affective and motivational appraisal process, was sensitive to counterfactual processing (Osinsky, Walter, & Hewig, 2014; Yeung & Sanfey, 2004). In the present study, after selecting between two gambles, the obtained outcome was presented, followed by the alternative outcome on the nonselected gamble. At the end of each trial, we asked participants to rate how pleased they were with the outcome. We hypothesized that the subjective ratings would be affected by both obtained and non-obtained outcomes. We also expected the facial muscle activity to be sensitive to counterfactual processes, such that corrugator response would be heightened for more positive non-obtained outcomes (i.e., regret), and that zygomaticus activity would be increased by more negative non-obtained outcomes (i.e., relief).

Third, to confirm the sensitivity of fEMG as an objective marker of emotional reactivity, we also presented affective images from the International Affective Picture System (IAPS) during a second task. Larsen et al. (2003) observed a strong linear effect of group-ranked valence on corrugator activity, such that the images ranked by the group as most aversive elicited greatest activity over this site. For the zygomaticus, a positive relationship was found with emotional valence, such that more positive stimuli evoked stronger muscle reactivity. Our second task was modeled precisely on that used by Larsen et al. (2003), in order to establish the fEMG responsivity for the decision task in the same participants.

## Method

### Participants

Fifty-one healthy, right-handed volunteers (26 males and 25 females; mean age = 24.5; *SD* = 4.2; age range = 19–35) were recruited from University of Cambridge. Participants completed the gambling task followed by the affective images task. One male participant did not complete the affective images task. The study was conducted in accordance with the Declaration of Helsinki and was approved by the University of Cambridge Psychology Research Ethics Committee. Written informed consent was obtained from all participants. Participants were paid a fixed fee as reimbursement for their time, plus a financial bonus that was proportional to their actual earnings in the gambling task.

### Gambling Task

**Figure 1:**
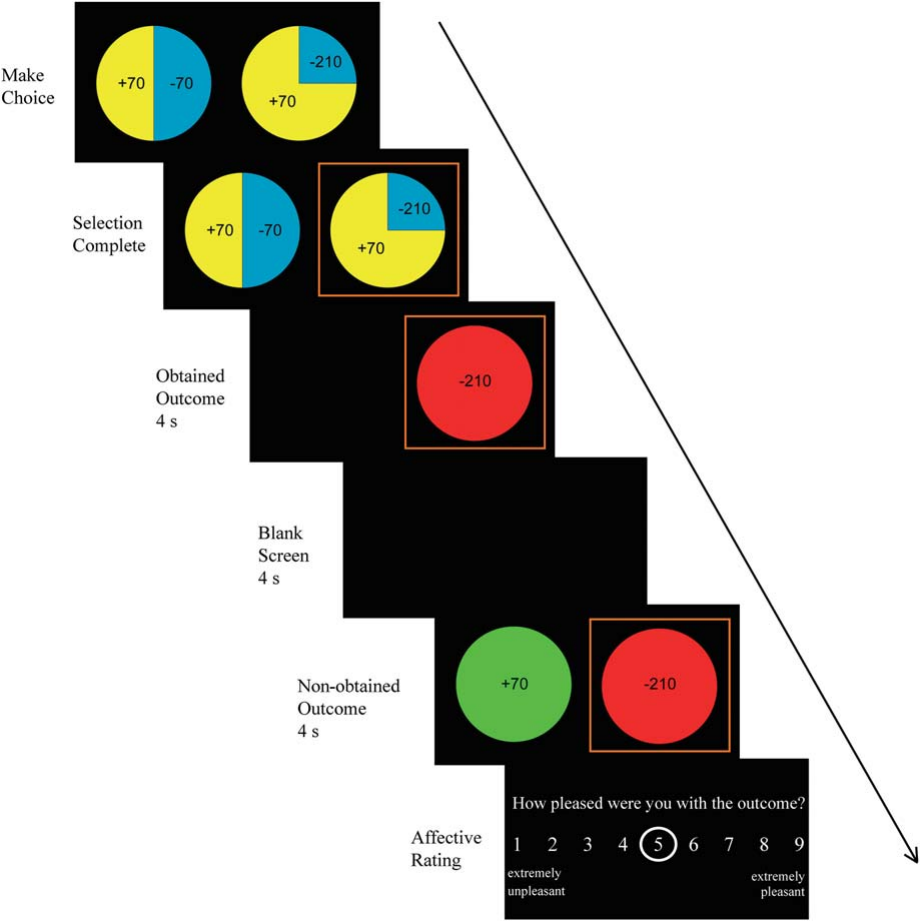
Sequence of events in a single trial in the gambling task. This trial displays a regret condition where the obtained outcome is more negative than non-obtained outcome on the nonselected wheel.

Participants performed 112 trials of a gambling task modified from Camille et al. (2010), which involved real monetary wins and losses. The task was programmed using Presentation software (Neurobehavioral System Inc.). On each trial, participants chose between two wheels that displayed different potential gains and losses, and their respective probabilities (see online supporting information for the full list of gambling pairings). Each wheel offered two of the following possible outcomes: +70, +210, −70, −210, representing monetary values in pence (i.e., British £). The outcome probabilities could be 0.25, 0.5, or 0.75, as indicated by the size of the segment (see Figure 1). As participants selected a wheel, it was highlighted with a red surround. Then, the outcome on the selected wheel (i.e., obtained outcome) was presented for 4 s, with the nonselected wheel covered. After a further 4 s of blank screen, the outcome on the nonselected wheel (i.e., non-obtained outcome) was presented alongside the obtained outcome for 4 s. Participants were then asked to rate, “How pleased were you with the outcome?” with 1 = *extremely unpleasant* and 9 = *extremely pleasant*. This was followed by a 4-s intertrial interval (ITI). No time constraints were imposed on wheel selection or affect ratings. Outcomes were prespecified to be in line with the displayed probabilities, ensuring that the task was fair. On average, participants won £12.65 (*SD* = 5.51) on the task.

### Affective Images Task

This task used the same IAPS stimuli and trial timings as Larsen et al. (2003). On each trial, a 3-s ITI was followed by the image being displayed for 6 s. After a blank screen for 3 s, two ratings were presented for valence (i.e., “How pleased were you with the picture?”) and arousal (i.e., “How excited were you by the picture?”), using 9-point Likert scales.

### Facial EMG Measurement

Facial EMG data were collected via a BIOPAC (Santa Barbara, CA, MP36R, recording at 1,000 samples per second. The BIOPAC was connected to a stimulus delivery computer and a second administrator computer running Acqknowledge v4.1. Events occurring on the stimulus delivery computer (including the outcomes on the task) were synchronized to the fEMG recording using digital channels. Facial EMG recordings were collected through 4-mm shielded chloride electrodes attached to the skin over the left eye (i.e., corrugator) and left cheek (i.e., zygomaticus) via 4-mm adhesive disks, according to the standard procedures established previously (Fridlund & Cacioppo, 1986). Following attachment of fEMG electrodes, 5 min of resting state data were acquired, before the instructions for the gambling task were read to the participant.

### Data Processing and Analysis

Data were screened prior to analysis and resampled at 100 Hz. The raw fEMG data, recorded at 5–500 Hz, were extracted using an inhouse script programmed in R Studio (R Development Core Team, 2008). The data were filtered through a 30 Hz high-pass filter to remove low frequency noise and artifacts recorded during the task. The filtered data were then rectified, converting negative values into positive values. For the gambling task, mean values were extracted for the final 1 s of the ITI as a baseline, and for the 4-s outcome period (time course data over the 4-s outcome period are presented in the supporting information). Similarly, for the affective images task, mean values were extracted for the 1 s prior to image onset as baseline, and for 6-s period that the image was displayed. For both tasks, the percentage change from baseline was used as the dependent variable.

We used R and *lme4* (Bates, Maechler, & Bolker, 2012) to perform a linear mixed effects analysis on the affect ratings, facial muscle response to obtained and non-obtained outcomes. We use linear mixed effects (LME) modeling via restricted maximum likelihood for all repeated measures analyses to reduce information loss when evaluating large, unbalanced data sets after signal standardization (Judd, Westfall, & Kenny, 2012). As a random effect, we had an intercept representing participant number. For affect ratings and facial muscle responses to counterfactual comparisons (i.e., when both obtained and non-obtained outcomes were presented), we looked at the effect of obtained and non-obtained outcomes (with its interaction term). Both obtained and non-obtained outcomes were treated as continuous fixed effect predictors. For facial muscle response to obtained outcomes, we assessed the impact of obtained outcomes. Visual inspection of residual plots did not reveal any obvious deviations from homoscedasticity or normality. *P* value was derived by the *lmerTest* package (Kuznetsova, Christensen, & Brockhoff, 2012).

For the fEMG data in the affective images task, we ranked averaged valence ratings across all the participants, and sorted EMG data based on these ranks (see Larsen et al., 2003). We tested the linear relationship between stimuli valence and the fEMG activity.

## Results

### Gambling Task

#### Affect ratings

**Figure 2:**
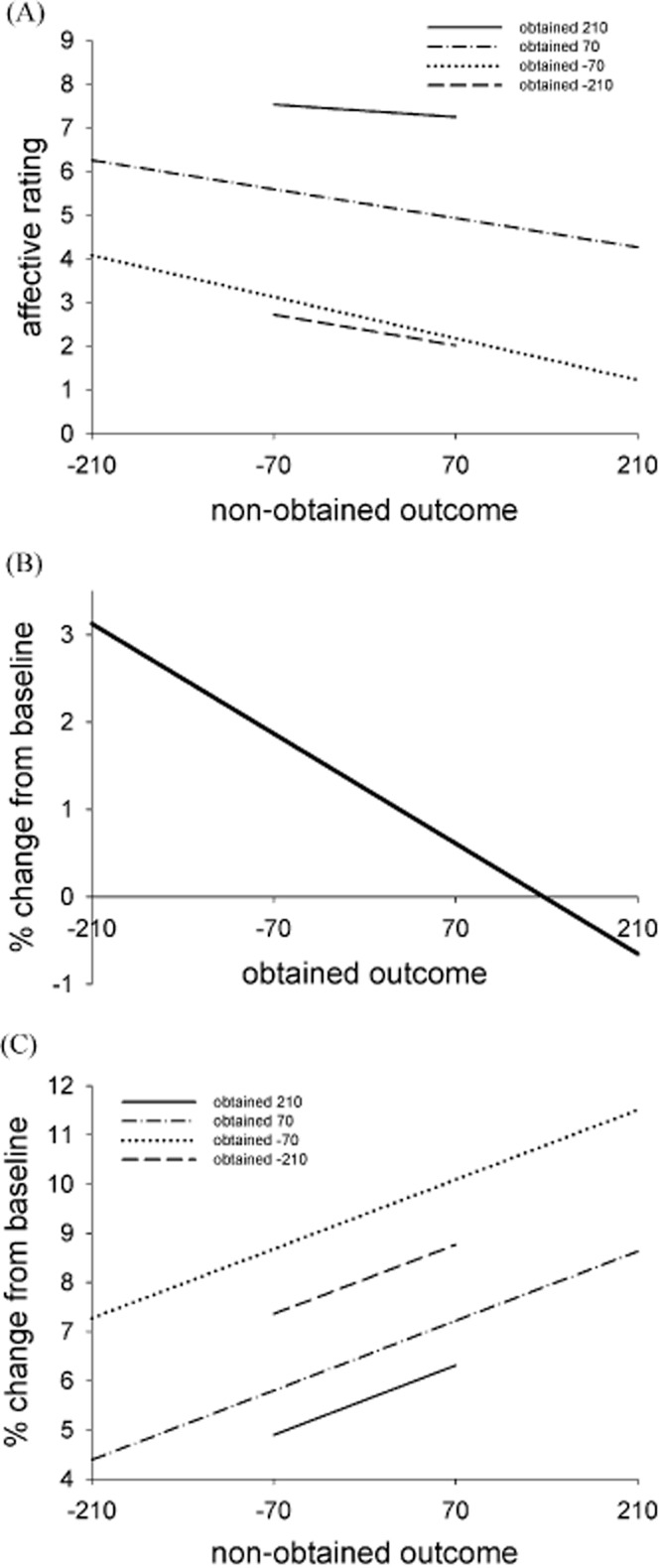
A: The effect of non-obtained outcome on affective ratings, at each level of objective outcome. B: The effect of obtained outcome on corrugator reactivity. C: The effect of non-obtained outcome on corrugator reactivity following the presentation of the counterfactual comparison, at each level of objective outcome. The fitted lines are derived from regression models.

Subjective ratings were analyzed with a model of Obtained Outcome × Non-obtained Outcome. This revealed a large and significant main effect of obtained outcome, *b* = 0.013, *SE* = 0.00015, *t* = 84.51, *p* < .001, with higher ratings following more positive obtained outcomes. There was also a significant main effect of non-obtained outcome, *b* = −0.0057, *SE* = 0.00014, *t* = −40.38, *p* < .001, due to higher ratings following more negative non-obtained outcomes. There was an interaction between obtained and non-obtained outcomes, *b* = −0.000013, *SE* = 0.0000017, *t* = −7.62, *p* < .001, which was decomposed by looking at the effect of non-obtained outcomes at each level of objective outcome (see Figure 2A). When participants objectively won the maximum amount (i.e., +210), they felt subjectively better if the non-obtained outcomes were more negative (i.e., relief), *b* = −0.002, *SE* = 0.0005, *t* = −4.39, *p* < .001. When they objectively won a moderate amount (i.e., +70), they felt worse if the non-obtained outcome was more positive (+210, i.e., regret) and felt better if the non-obtained outcome was more negative (−70 or −210, i.e., relief), *b* = −0.005, *SE* = 0.0001, *t* = −32.55, *p* < .001. This slope was steepest when participants objectively lost a moderate amount (i.e., −70), *b* = −0.007, *SE* = 0.0002, *t* = −36.27, *p* < .001, in which case they felt better if the non-obtained outcome was −210, and felt worse if the non-obtained outcome was +70 or +210. When participants lost the maximum amount (i.e., −210), they felt worse if the non-obtained outcomes were more positive (i.e., regret), *b* = −0.005, *SE* = 0.001, *t* = −3.88, *p* < .001, and it looks as though this effect was blunted due to a floor effect.

#### Facial muscle responses to obtained outcomes

The corrugator scaled with the magnitude of the objective outcome, *b* = −0.009%, *SE* = 0.00003, *t* = −2.76, *p* < .01, with more negative outcomes heightening corrugator responses (i.e., disappointment; see Figure 2B; see Figure S1 in the supporting information for the time course data). The zygomaticus did not vary linearly with objective outcome, *p* > .1.

#### Facial muscle responses to counterfactual comparisons

In the model looking at the fEMG responses to the presentation of the counterfactual comparisons, the significant main effect of the obtained outcome was corroborated, *b* = −0.01%, *SE* = 0.00004, *t* = −2.86, *p* < .01, with more negative obtained outcomes eliciting stronger corrugator responses (see Figure 2C; see Figure S2 in the supporting information for time course data). There was also a significant main effect of non-obtained outcome, *b* = 0.01%, *SE* = 0.00004, *t* = 2.68, *p* < .01, with more positive non-obtained outcomes (i.e., regret) heightening corrugator reactivity. The interaction between obtained and non-obtained outcome was not significant, *p* > .1. For the model on zygomaticus reactivity, there were no significant main effects of obtained or non-obtained outcomes, nor an interaction effect, *p*s > .1.

In light of previous work showing that event-related potentials associated with outcome evaluation and reward processing are sensitive to outcome probability and unexpectedness (Hajcak, Holroyd, Moser, & Simons, 2005; Hajcak, Moser, Holroyd, & Simons, 2007; Wu & Zhou, 2009), we tested for these effects in our fEMG data by introducing predictors for the obtained and non-obtained outcome probabilities (plus their interaction terms) into the statistical models. Neither the main effects nor interaction terms for the probability predictors were significant, *p*s > .1.

### Affective Images Task

**Figure 3:**
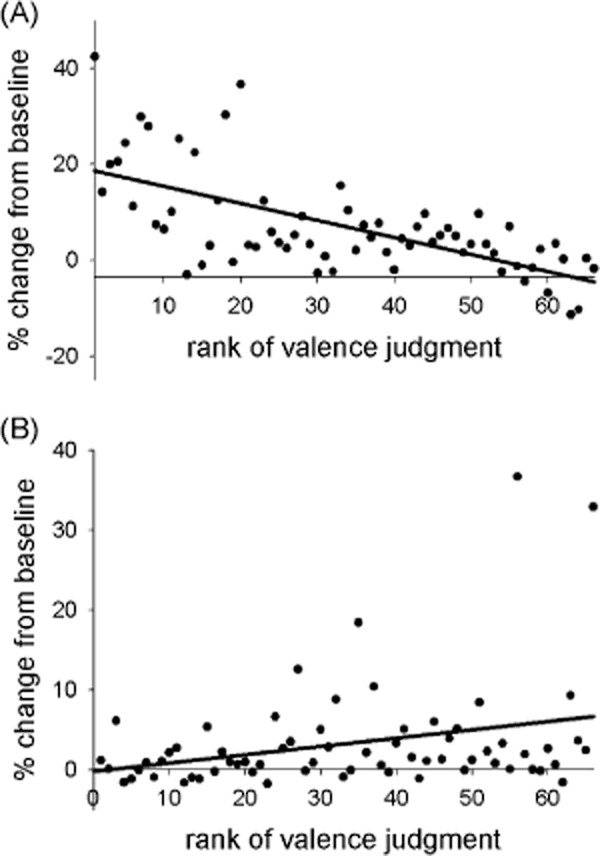
Psychophysiological activity during the affective images task. A: Corrugator reactivity by ranked stimulus valence. B: Zygomaticus reactivity by ranked stimulus valence.

There was a strong negative relationship between stimuli valence and corrugator reactivity, such that more negative pictures elicited stronger responses (see Figure 3A), *b* = −0.3%, *SE* = 0.0003, *t* = −11.46, *p* < .001. Zygomaticus reactivity showed a significant positive relationship with stimuli valence such that more positive pictures evoked stronger responses (see Figure 3B), *b* = 0.1%, *SE* = 0.0004, *t* = 2.53, *p* = .01.

## Discussion

Using a gambling task, our first observation was that fEMG corrugator responses scaled negatively with the magnitude of the obtained outcomes, with the greatest responses to large losses (i.e., disappointment). This is one of the first studies to characterize fEMG responses to decision outcomes, and the first demonstration of corrugator activity to financial losses (cf. Bediou et al., 2011). In our gambling task, we also displayed the non-obtained outcome on the gamble wheel that was rejected, at an 8-s delay after the obtained outcome. Emotional self-report ratings at the end of each trial were sensitive to the magnitude of both the obtained and the non-obtained outcomes (Camille et al., 2004, 2010; Coricelli et al., 2005), confirming that the task successfully induced counterfactual thinking. Our participants reported lower emotional ratings as the non-obtained outcomes were increasingly positive, consistent with regret, and they reported higher emotional ratings when non-obtained outcomes were more negative, consistent with relief. Corrugator activity was also sensitive to these counterfactual comparisons, such that its activity scaled positively with the magnitude of the non-obtained outcomes. This finding extends previous reports that electrodermal activity (EDA) is also increased when the non-obtained outcome exceeds the obtained outcome (i.e., the regret condition; Camille et al., 2004; Chandrasekhar, Capra, Moore, Noussair, & Berns, 2008). Given that EDA is a marker of general arousal, we argue that the corrugator response constitutes a clearer physiological counterpart to the evoked negative affect.

Given its established role in positive affect, we predicted that zygomaticus activity would be positively related to the magnitude of obtained outcomes and negatively related to non-obtained outcomes (i.e., activated during relief). Neither prediction was supported, and recording at the zygomaticus site did not show any significant fluctuation during the gambling task. These results fail to corroborate a previous study by Bediou et al. (2011) in which zygomaticus activity was greater following financial gains compared to financial losses in a social competition task. In this regard, it is important that our participants also completed an affective images task during the same session, and we were able to replicate the established profile of increasing zygomaticus activity with self-reported appetitive ratings to IAPS pictures (see Lang et al., 1993; Larsen et al., 2003). As such, our null findings in the gambling task cannot be readily attributed to electrode placement or poor data quality, as we have evident fEMG sensitivity to the IAPS pictures. On the affective images task, we also replicated the typical corrugator profile, such that negative pictures elicited stronger activity, and as a function of self-reported valence ratings.

For the zygomaticus response, past studies have reported a quadratic relationship between stimuli valence and zygomaticus reactivity, such that activity is increased following intensely positive but also intensely negative stimuli. We tested for a quadratic function with gambling outcomes, and there was again no significant relationship, *p* > .1. We note that past work has tended to show stronger effect sizes for corrugator activity compared to zygomaticus activity, and this is likely due to physiological differences between these two facial muscle sites (i.e., recording on the brow compared to recording on the cheek; Bradley & Lang, 2000; Larsen et al., 2003). For example, emotional auditory clips influenced corrugator responses in a negative linear manner, but did not affect zygomaticus activity (Bradley & Lang, 2000).

The asymmetrical sensitivity of the corrugator and zygomaticus on the gambling task may alternatively reflect the basic properties of human decision making. It is well established that potential losses have a greater impact upon human choice than equivalently sized gains (“loss aversion,” Tversky & Kahneman, 1981), and realized negative outcomes also tend to be processed more thoroughly than positive ones (Baumeister, Bratslavsky, Finkenauer, & Vohs, 2001). It is also possible that the corrugator may show greater sensitivity to immediate feedback and automatic affective processes compared to the zygomaticus (see supporting information for the time course of fEMG). Future studies could benefit from directly comparing the time course of facial muscle responses to affective stimuli across different muscle sites. We would encourage further research characterizing the boundary conditions for zygomaticus reactivity during decision-making tasks, and this work may fruitfully utilize fEMG in more naturalistic settings.

Psychophysiological experiments can directly inform a long-standing debate in decision-making research as to the role of emotional responses in contributing to “rational” choice (Damasio, 2008; see Clark et al., 2012; Studer & Clark, 2011). The phenomenon of regret is a compelling case of how an intense emotion can be induced by observing the outcomes of rejected options. As humans, we learn to make decisions that avoid experiencing such negative states (Bell, 1982; Camille et al., 2004; Coricelli et al., 2005). While counterfactual thinking can be reduced to the cognitive (i.e., “cold”) comparison of the obtained against the non-obtained outcome, the present demonstration of corrugator reactivity in the regret condition adds to other lines of evidence from functional imaging and neuropsychology that this is an inherently emotional process. These findings highlight the utility of facial EMG, in particular the corrugator supercilii, as an objective marker of emotional reactivity in decision-making and gambling studies.

## Supporting Information

**Appendix S1:** Parameters for the gambling task. **Figure S1:** Time course of corrugator reactivity to obtained outcome. **Figure S2:** Time course of corrugator reactivity to counterfactual comparison. **Figure S3:** Time course of zygomaticus reactivity to obtained outcome. **Figure S4:** Time course of zygomaticus reactivity to counterfactual comparison.
