# Supplementary material for: Disappointment and regret enhance corrugator reactivity in a gambling task
Source: Psychophysiology. 2014 Oct 24;52(4):518–23. doi: 10.1111/psyp.12371 (PMC4510786; doi:10.1111/psyp.12371)

**Supporting Information, Wu & Clark “Disappointment and regret enhance corrugator reactivity in a gambling task”**

**Appendix S1: Parameters for the gambling task**


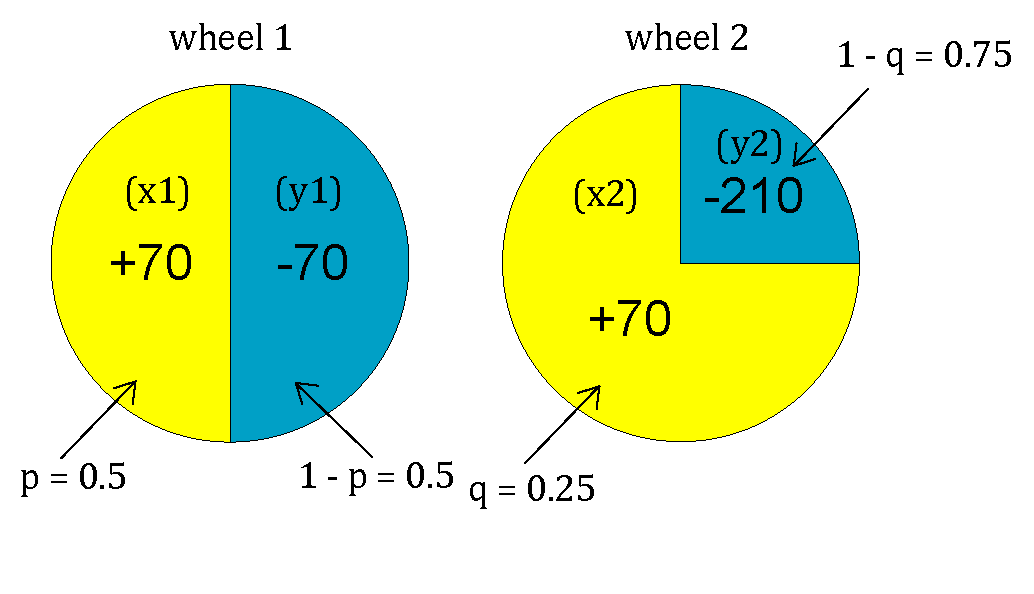


Outcome x is always more positive (x1 > y1 and x2 > y2)

| wheel 1 wheel 2 | | | | | | | | | |
| --- | --- | --- | --- | --- | --- | --- | --- | --- | --- |
| x1 | p | y1 | 1-p | x2 | q | y2 | 1-q | number of trials |  |
| 70 | 0.5 | -70 | 0.5 | 210 | 0.25 | -70 | 0.75 | 24 |  |
| 70 | 0.5 | -70 | 0.5 | 70 | 0.75 | -210 | 0.25 | 24 |  |
| 70 | 0.25 | -70 | 0.75 | 210 | 0.75 | -70 | 0.25 | 8 |  |
| 70 | 0.75 | -70 | 0.25 | 70 | 0.25 | -210 | 0.75 | 8 |  |
| 210 | 0.25 | -70 | 0.75 | 70 | 0.5 | -70 | 0.5 | 24 |  |
| 70 | 0.75 | -210 | 0.25 | 70 | 0.5 | -70 | 0.5 | 24 |  |

Participants were presented with 112 choice trials between two wheels depicting gains and their respective probabilities. The outcomes were pre-specified to be consistent with the displayed probabilities. There were 12 possible obtained and non-obtained outcome pairs {-210, -70; -210, 70; -70, -210; -70, -70; -70, 70; -70, 210; 70, -210; 70, -70; 70, 70; 70, 210; 210, -70; 210, 70}.

**Figure S1.** Time course of corrugator reactivity to obtained outcome.





**Figure S2.** Time course of corrugator reactivity to counterfacutal comparison. A: when the obtained outcome was +210. B: when the obtained outcome was +70. C: when the obtained outcome was -70. D: when the obtained outcome was -210.


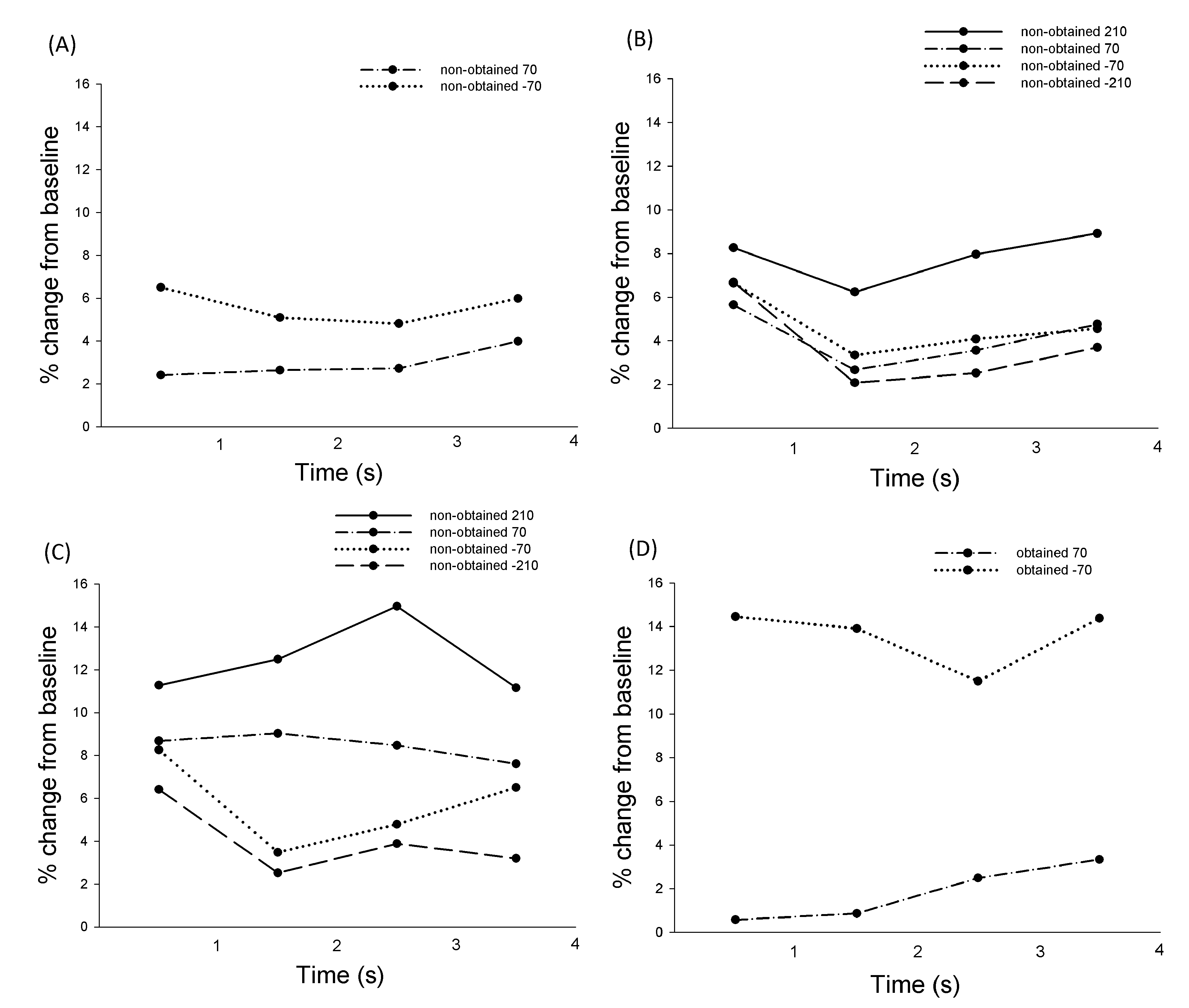


**Figure S3.** Time course of zygomaticus reactivity to obtained outcome.





**Figure S4.** Time course of zygomaticus reactivity to counterfacutal comparison. A: when the obtained outcome was +210. B: when the obtained outcome was +70. C: when the obtained outcome was -70. D: when the obtained outcome was -210.


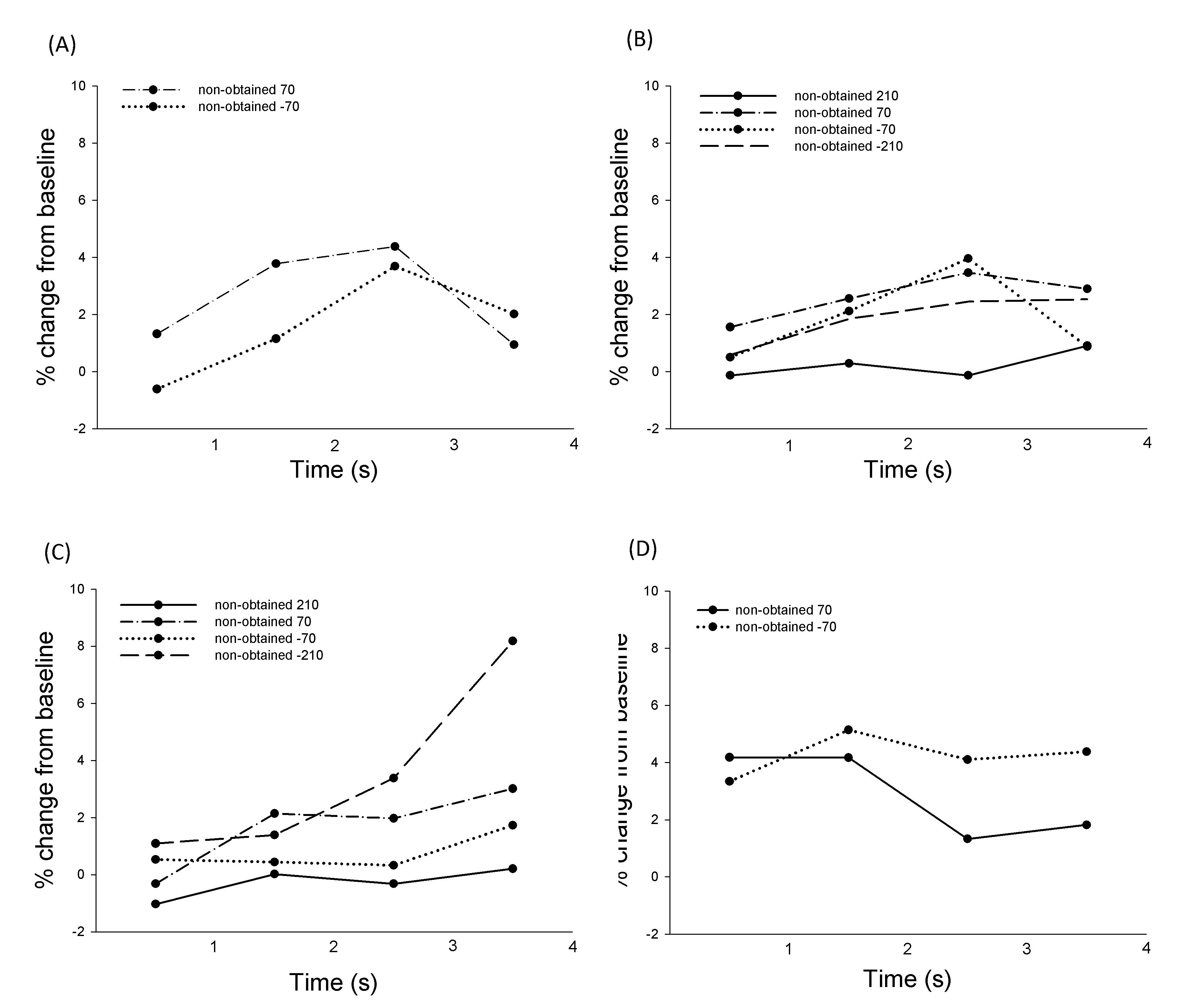

Supplement: Supplementary file 1 [file psyp0052-0518-sd1.docx]
